# Absorption of Fat

**Published:** 1890-07-05

**Authors:** 


### ABSORPTION OF FAT.

Minkowski found that after extirpation of the pancreas animals entirely lost the power of absorbing fat, except in the form of milk, though if pig's pancreas were mixed with the food the fat was digested and retained. He therefore concludes that the secretion of the pancreas is, if not the sole, at any rate an essential factor in the absorption of fat, reducing it to an emulsion or a state of physical and mechanical division and suspension, similar to that in which it exists in milk.

